# Product Profiles of Promiscuous Enzymes Can be Altered by Controlling In Vivo Spatial Organization

**DOI:** 10.1002/advs.202303415

**Published:** 2023-09-26

**Authors:** Li Chen Cheah, Lian Liu, Manuel R. Plan, Bingyin Peng, Zeyu Lu, Gerhard Schenk, Claudia E. Vickers, Frank Sainsbury

**Affiliations:** ^1^ Australian Institute for Bioengineering and Nanotechnology The University of Queensland St Lucia QLD 4072 Australia; ^2^ CSIRO Future Science Platform in Synthetic Biology Commonwealth Scientific and Industrial Research Organisation (CSIRO) Dutton Park St Lucia QLD 4102 Australia; ^3^ Metabolomics Australia (Queensland Node) The University of Queensland St Lucia QLD 4072 Australia; ^4^ ARC Centre of Excellence in Synthetic Biology Queensland University of Technology Brisbane QLD 4000 Australia; ^5^ School of Biological and Environmental Science Queensland University of Technology Brisbane QLD 4000 Australia; ^6^ School of Chemistry and Molecular Biosciences The University of Queensland St Lucia QLD 4072 Australia; ^7^ Centre for Cell Factories and Biopolymers Griffith Institute for Drug Discovery Griffith University Nathan QLD 4111 Australia; ^8^ Present address: Australian Centre for Disease Preparedness 5 Portarlington Rd East Geelong VIC 3219 Australia

**Keywords:** encapsulation, metabolic engineering, promiscuous enzyme, terpenoid, virus‐like particles

## Abstract

Enzyme spatial organization is an evolved mechanism for facilitating multi‐step biocatalysis and can play an important role in the regulation of promiscuous enzymes. The latter function suggests that artificial spatial organization can be an untapped avenue for controlling the specificity of bioengineered metabolic pathways. A promiscuous terpene synthase (nerolidol synthase) is co‐localized and spatially organized with the preceding enzyme (farnesyl diphosphate synthase) in a heterologous production pathway, via translational protein fusion and/or co‐encapsulation in a self‐assembling protein cage. Spatial organization enhances nerolidol production by ≈11‐ to ≈62‐fold relative to unorganized enzymes. More interestingly, striking differences in the ratio of end products (nerolidol and linalool) are observed with each spatial organization approach. This demonstrates that artificial spatial organization approaches can be harnessed to modulate the product profiles of promiscuous enzymes in engineered pathways in vivo. This extends the application of spatial organization beyond situations where multiple enzymes compete for a single substrate to cases where there is competition among multiple substrates for a single enzyme.

## Graphical abstract

To explore the potential role of spatial organization in regulating promiscuous metabolic enzymes, a terpene synthase is co‐localized with the preceding enzyme in the pathway by translational fusion and/or co‐encapsulation in a self‐assembling protein cage. Compared to unorganized enzymes, in vivo spatial organization increases product titers and alters the ratio of the two terpenoid end products.

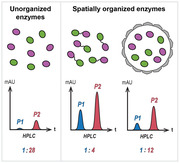

## 1 Introduction

Hundreds of metabolic reactions are coordinated simultaneously in a cell. A recurring metabolic control strategy in nature is to spatially organize the enzymes that participate in a reaction cascade using metabolons (transient protein complexes) or cellular compartments. Inspired by natural systems, enzyme spatial organization has been implemented in heterologous bioproduction pathways using strategies such as translational protein fusion,^[^ ^1^, ^2^ ^]^ interactions with synthetic scaffolds,^[^ ^3^, ^4^, ^5^ ^]^ and sequestration within membrane‐bound organelles.^[^ ^6^, ^7^, ^8^ ^]^ More recently, self‐assembling protein compartments such as virus‐like particles (VLPs),^[^ ^9^, ^10^, ^11^, ^12^ ^]^ bacterial microcompartments,^[^ ^13^, ^14^, ^15^, ^16^ ^]^ and encapsulins^[^ ^17^, ^18^ ^]^ have been used to encapsulate metabolic enzymes in vivo. Despite their size and apparent complexity, these supramolecular structures self‐assemble from only a few types of repeating building blocks, which allows them to be easily expressed in heterologous hosts.

Historically, the primary goal of introducing enzyme spatial organization has been to enhance metabolic flux through a multi‐enzyme cascade and reduce crosstalk with competing pathways.^[^ ^19^, ^20^, ^21^, ^22^ ^]^ However, other biocatalytic parameters are also of interest to metabolic engineers. In particular, some enzymes lack product specificity, decreasing the efficiency of production. There are some examples that suggest that modifying spatial organization could also alter product specificity in promiscuous enzymes. When geranyl diphosphate synthase and pinene synthase were expressed in *E. coli*, different ratios of the two terpenoid products (i.e., α‐ and β‐pinene) were produced when the enzymes were expressed individually or as a translationally fused protein.^[^ ^23^ ^]^ The relocation of several enzymes in a recursive alcohol elongation pathway from the yeast cytoplasm to the mitochondrion caused an increase in the ratio of isopentanol (C5) to isobutanol (C4) produced.^[^ ^8^ ^]^ The co‐encapsulation of carbonyl reductase and glucose dehydrogenase in bacteriophage P22 VLPs enhanced enzyme stereoselectivity, increasing the enantiomeric excess of chiral benzylic alcohol synthesis by up to 13% compared to unencapsulated enzymes.^[^ ^24^ ^]^ These observations mirror the natural role of metabolons in regulating the promiscuous enzymes of the tricarboxylic acid cycle (Krebs cycle).^[^ ^25^, ^26^ ^]^ These examples also point toward broader applications of spatial organization for modulating enzyme behavior in engineered biosynthetic pathways.

Substrate promiscuity is widespread in enzymes involved in plant specialized metabolism (previously called “secondary metabolism”), such as terpene synthases.^[^ ^27^, ^28^ ^]^ Terpene synthases are industrially relevant enzymes, synthesizing the largest and most diverse class of natural products, that is, terpenoids.^[^ ^29^ ^]^ The massive diversity in terpenoid chemistry is thought to be driven by the broad promiscuity of terpene synthases, which enables the enzymes to adapt to new substrates in response to evolutionary pressures.^[^ ^28^, ^30^, ^31^ ^]^ This promiscuity has been exploited to access novel chemistries since terpene synthases can often accommodate a range of non‐native and even non‐natural substrates.^[^ ^32^, ^33^ ^]^

Promiscuous enzymes need to be carefully regulated to minimize unproductive reactions and potentially toxic by‐products.^[^ ^26^ ^]^ The examples described above suggest that for promiscuous enzymes that participate in a reaction cascade, the substrate preference could be controlled through enforcing spatial organization. The co‐localization of a promiscuous enzyme with the preceding enzyme in the pathway could limit the entry of non‐target substrates, avoiding wasteful catalytic conversions. It has been shown that metabolon formation is a widespread strategy in land plants for managing enzyme promiscuity in flavonoid biosynthesis.^[^ ^27^, ^34^ ^]^ Together, these observations compelled us to ask if substrate use and product ratios could be controlled in promiscuous enzymes in vivo using precise artificial spatial organization in an engineered bioproduction pathway. The capability to tune the substrate preference would both increase the efficiency of production and reduce the requirement for further downstream purification.

To examine spatial organization as an orthogonal mechanism to control product profiles, we used co‐encapsulation in VLPs and/or translational protein fusion of a promiscuous terpene synthase, nerolidol synthase (NES), in budding yeast (*Saccharomyces cerevisiae*) in vivo. This enzyme can accept both C10 (geranyl diphosphate, GPP) and C15 (farnesyl diphosphate, FPP) substrates, producing linalool and nerolidol, respectively. NES was co‐encapsulated and/or fused with farnesyl diphosphate synthase (FPPS), the preceding enzyme in the pathway. We show that each spatial organization approach led to different ratios of the two terpenoid end products (nerolidol and linalool), demonstrating that the product ratio of promiscuous enzymes can be modulated by controlling in vivo spatial organization.

## 2 Results and Discussion

### 2.1 Extending a Nanocage Platform for Dual Protein Co‐Encapsulation

**Figure 1:**
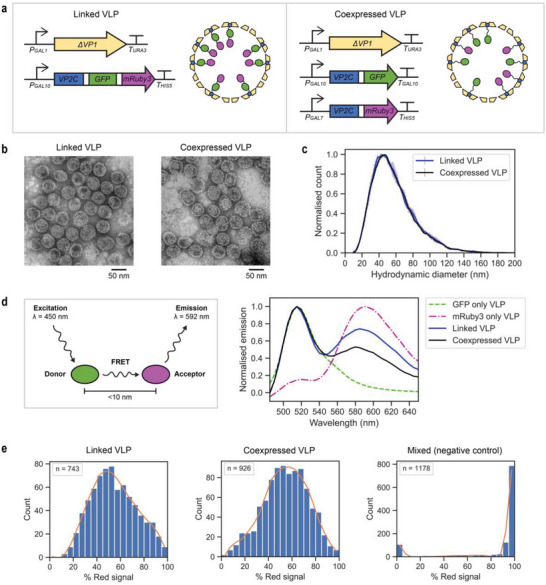
Characterization of the MPyV VLP platform. a) Schematic of the “Linked VLP” and “Coexpressed VLP” cargo co‐encapsulation strategies. Green and red fluorescent proteins (GFP and mRuby3) were used as model cargo. Each domain in the fusion proteins was connected by flexible linkers (shown in white). Representations are not drawn to scale. b) Negatively stained transmission electron micrographs of purified VLPs. c) Hydrodynamic diameter distributions of purified VLPs, measured using nanoparticle tracking analysis. The mean of three technical replicates is shown, with error bars indicating +/− 1 standard deviation. d) Förster resonance energy transfer (FRET) assay, with GFP as the donor and mRuby3 as the acceptor. The mean spectra of three technical replicates are shown. e) Semi‐quantitative analysis of GFP and mRuby3 co‐localization by super‐resolution structured illumination microscopy (SR‐SIM). The histograms show the proportion of red signal (from mRuby3) to the total signal (Green + Red) from each individual particle. Histograms were plotted with bin width = 5, and the number of particles in the dataset (n) is shown as an inset.

We have previously developed an in vivo‐assembling nanocage platform for *S. cerevisiae* based on murine polyomavirus (MPyV) VLPs.^[^ ^11^ ^]^ Each VLP shell is nominally composed of 360 copies of the major capsid protein VP1, which are arranged in 72 pentamers.^[^ ^35^ ^]^ In the MPyV VLP system, specific encapsulation of cargo proteins occurs by tagging the protein of interest with an N‐terminal anchor called VP2C.^[^ ^11^, ^36^ ^]^ We first extended the VLP construct design for dual protein co‐encapsulation in vivo using green and red fluorescent proteins (GFP and mRuby3) to verify that tagged cargo proteins are appropriately co‐encapsulated. Cargo proteins were either tethered end‐to‐end (“Linked VLP” construct) or individually fused to the VP2C anchor (“Coexpressed VLP” construct); schematic diagrams of the expression strategies are shown in **Figure** **1**a. For Coexpressed VLP, different promoters were used for each VP2C‐cargo fusion to avoid inadvertent homologous recombination between cassettes.^[^ ^37^ ^]^

Both the Linked VLP and Coexpressed VLP strategies produced uniform particles of similar morphology under transmission electron microscopy (Figure 1b), which also closely resemble MPyV VLPs that encapsulate GFP alone.^[^ ^11^ ^]^ Nanoparticle tracking analysis shows nearly identical distributions of the hydrodynamic diameter (Figure 1c), indicating that the configuration of cargo proteins did not affect the assembly and morphology of the nanocage in vivo. Each construct resulted in encapsulation of both fluorescent protein cargos and cargo loading estimated by SDS‐PAGE (Figure S1, Supporting Information) gave ≈72 units of VP2C‐GFP‐mRuby3 per Linked VLP (full theoretical capacity) and ≈61 units of either VP2C‐GFP or VP2C‐mRuby3 per Coexpressed VLP.

Ensemble measurement techniques cannot determine if the two cargo proteins were successfully co‐encapsulated within individual VLPs. We thus examined if co‐encapsulated fluorescent proteins are in close proximity using Förster resonance energy transfer (FRET). For fluorescent proteins, efficient energy transfer only occurs if the donor and acceptor are within ≈10 nm.^[^ ^38^ ^]^ Co‐encapsulation of fluorescent protein cargos within VLP‐based nanocages brings them to within this range, enabling FRET.^[^ ^12^, ^36^ ^]^ Accordingly, the emission spectra of both constructs under donor excitation show a clear FRET acceptor peak in the mRuby3 emission range (Figure 1d). Control VLPs with either GFP or mRuby3 alone did not show a significant FRET peak with the same settings (Figure S2, Supporting Information). Moreover, mixing the control VLPs did not increase the signal in the acceptor range, indicating that co‐encapsulation is required for energy transfer to occur (Figure S2, Supporting Information). Coexpressed VLP exhibited slightly lower FRET than Linked VLP, presumably due to an uneven distribution of GFP and mRuby3 between and within VLPs. The distance between cargo proteins tethered to adjacent pentamers is estimated to be ≈6.9 nm.^[^ ^36^ ^]^ However, this is likely to be an underestimate of the actual average distance between donor and acceptor in Coexpressed VLP because VLPs are only partially filled. In contrast, translationally fused GFP and mRuby3 (in Linked VLP) is spaced ≈5.2 nm apart, assuming a contour length of 4.0 Å per residue^[^ ^39^ ^]^ for the 13‐residue linker peptide; the average residence distance is likely to be even smaller since the linker is flexible.

The degree of GFP and mRuby3 co‐encapsulation in individual VLPs was measured semi‐quantitatively using a single particle imaging technique, super‐resolution structured illumination microscopy (SR‐SIM) (Figure 1e; Figures S3–S5, Supporting Information). A similar SR‐SIM protocol was previously used to examine in vitro‐assembled MPyV VLPs.^[^ ^36^ ^]^ The red fluorescence signal of each particle was divided by the total signal intensity (green + red) of that particle, providing a measure of the mRuby3:GFP ratio. Both Linked VLP and Coexpressed VLP produced approximately normal distributions of the mRuby3:GFP ratio (represented as % Red signal; Figure 1e), indicating effective fluorescent protein co‐encapsulation. This was not observed in the negative control sample (mixed GFP‐only and mRuby3‐only VLPs), confirming that co‐localization was within particles and not due to low‐order aggregates of VLPs.

### 2.2 Spatial Organization of Promiscuous Metabolic Enzymes Alters the Product Profile

**Figure 2:**
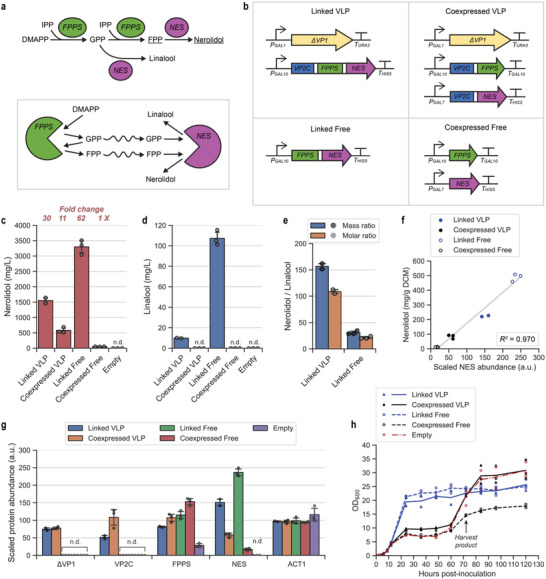
Spatial organization of farnesyl diphosphate synthase (FPPS) and nerolidol synthase (NES). a) Reactions catalyzed by FPPS and NES, two successive, promiscuous enzymes in a terpenoid pathway. The major products of FPPS and NES are FPP and nerolidol respectively (underlined). b) Expression cassettes for the four enzyme configurations in this study. c) Nerolidol titers at 72 h. “Fold change” indicates the relative titer compared to the Coexpressed Free strain. “Empty” is the base strain transformed with an empty vector. d) Linalool titers at 72 h. e) Mass ratios and molar ratios of the NES products nerolidol and linalool. f) Relationship between nerolidol production and relative NES expression. Fitting a linear regression model using the least squares method produces *R^2^* = 0.970. g) Relative in vivo levels of key proteins in the engineered nerolidol pathway at 72 h. ACT1 (actin) is included as a housekeeping reference protein. h) Cell density (measured using OD_600_) over the course of fermentation. For each chart, means and individual data points are shown. Error bars are +/−1 STD. “n.d.” = not detected.

The mevalonate pathway produces the universal terpenoid precursors isopentenyl diphosphate (IPP) and dimethylallyl diphosphate (DMAPP). These precursors are successively condensed into the longer chain intermediates geranyl diphosphate (GPP) and farnesyl diphosphate (FPP) by yeast FPP synthase (FPPS, also called ERG20) (**Figure** **2**a).

Nerolidol is a sesquiterpene with applications as a perfume and flavor additive^[^ ^40^ ^]^ and conventional methods to produce it have poor yields.^[^ ^40^ ^]^ Nerolidol synthase (NES) from *Actinidia chinensis* (golden kiwifruit) is promiscuous; besides its main activity of converting FPP (C15) into nerolidol (C15), it can also weakly convert GPP (C10) into linalool (C10)^[^ ^41^ ^]^ (Figure 2a). We wanted to examine if spatial organization of NES and the preceding enzyme in the pathway, FPPS, could alter the ratio of the end products nerolidol and linalool. The promiscuity of this NES variant provides an interesting opportunity to investigate the competition of multiple substrates for a single enzyme, which has never been studied using artificial spatial organization. NES is also expected to be one of the rate‐limiting enzymes of the pathway – since terpene synthases are kinetically “slow” enzymes^[^ ^42^, ^43^ ^]^ – maximizing the chance of observing an effect with spatial organization.

FPPS and NES were co‐encapsulated as for the fluorescent proteins above (Linked VLP and Coexpressed VLP) (Figure 2b). VLP assembly in these strains was confirmed by transmission electron microscopy (Figure S6, Supporting Information). In addition, the enzymes were also expressed as individual proteins (Coexpressed Free) or as an unencapsulated fusion protein (Linked Free)^[^ ^44^ ^]^ (Figure 2b). The native copy of the yeast FPPS gene was retained in all strains.

Spatial organization of FPPS and NES by VLP co‐encapsulation and/or translational fusion greatly enhanced terpenoid bioproduction compared to the Coexpressed Free strain – nerolidol titers were increased by ≈11‐fold (Coexpressed VLP), ≈30‐fold (Linked VLP), and ≈62‐fold (Linked Free) (Figure 2c). “Flux push” from the augmented mevalonate pathway in the base strain^[^ ^45^ ^]^ combined with possible “flux pull” from enzyme co‐localization resulted in exceptionally high product titers, even without optimized expression levels or fermentation conditions. We have previously reported nerolidol titers up to 4.2 g L^−1^ using a similar FPPS‐NES fusion construct.^[^ ^44^ ^]^ For comparison, the current highest titer reported for nerolidol bioproduction is 3.3 g L^−1^ in shake flask culture and ≈16 g L^−1^ using fed‐batch fermentation in *E. coli*, after extensive pathway optimization and genome editing.^[^ ^46^ ^]^ To our knowledge, the current work is the first demonstration of metabolic enzyme co‐encapsulation in a self‐assembling synthetic yeast compartment and represents the largest titer fold increases reported for engineered in vivo compartmentalization in yeast. The levels of squalene, ergosterol, and prenyl alcohols (geraniol, farnesol, and geranylgeraniol) indicated minimal metabolite drain into side reactions and the native ergosterol pathway (Figure S7, Supporting Information).

The ability of NES to use GPP as an alternative substrate, producing linalool, creates competition with FPPS at the GPP node (Figure 2a). While linalool production was not detected for the two constructs with unfused enzymes (Coexpressed VLP and Coexpressed Free), the FPPS‐NES fusion constructs Linked VLP and Linked Free produced substantial amounts of linalool (Figure 2d). This suggests that when translationally fused, NES can compete effectively with FPPS for GPP – this is remarkable considering that yeast FPPS is known to release only a small proportion of the GPP it produces.^[^ ^47^, ^48^ ^]^ There were striking differences in the ratio of nerolidol and linalool, where Linked VLP was about fivefold more selective for nerolidol production than Linked Free (Figure 2e).

We performed whole‐cell proteomics on samples collected at 72 h post‐inoculation to examine if the product titers could be attributed to differences in intracellular FPPS and NES levels. Nerolidol production roughly correlates with NES accumulation (Figure 2f), which is unsurprising given that terpene synthases are often the rate‐limiting enzyme in terpenoid production.^[^ ^42^, ^43^ ^]^ There was a >3‐fold higher level of NES in Coexpressed VLP compared to Coexpressed Free, despite being fused to the VP2C anchor that is known to destabilize cargo proteins in the absence of VLP assembly.^[^ ^11^ ^]^ A C‐terminally truncated version of ΔVP1^[^ ^49^ ^]^ that binds VP2C but does not self‐assemble into VLPs^[^ ^50^ ^]^ fails to stabilize cargo proteins in vivo (Figure S8, Supporting Information), providing supporting evidence that NES was stabilized by VLP encapsulation. Introduction of an extra copy of ΔVP1 to the Linked VLP and Coexpressed VLP strains did not further increase nerolidol or linalool titers (Figure S9, Supporting Information), suggesting that VLP capacity is not a limiting factor in this system.

Proteomics also revealed that one clone of the Linked VLP strain had anomalously elevated expression of the engineered nerolidol pathway (Figure S10, Supporting Information). Quantitative PCR on yeast genomic DNA confirmed that this clone contained two integrated copies of the entire nerolidol expression cassette (Figure S12, Supporting Information). Therefore, data from this clone has been omitted from the dataset presented in Figure 2 (refer to Figure S7, Supporting Information, for data including the anomalous clone).

Consistent with our previous work,^[^ ^44^ ^]^ the Coexpressed Free and empty vector controls showed impaired growth in the early phase of fermentation (Figure 2h), possibly from the toxic accumulation of terpenoid pathway intermediates.^[^ ^51^, ^52^ ^]^ FPPS and NES co‐localization by protein fusion was able to partly alleviate this toxicity, resulting in relatively normal growth of the Linked strains. Coexpressed VLP and the empty vector strain exhibited unusual growth profiles where growth was initially slow but recovered after 72 h, which suggests relief of the metabolic flux imbalance later in the fermentation.

### 2.3 Choice of Enzyme Configuration Differentially Modulates the Product Profile

**Figure 3:**
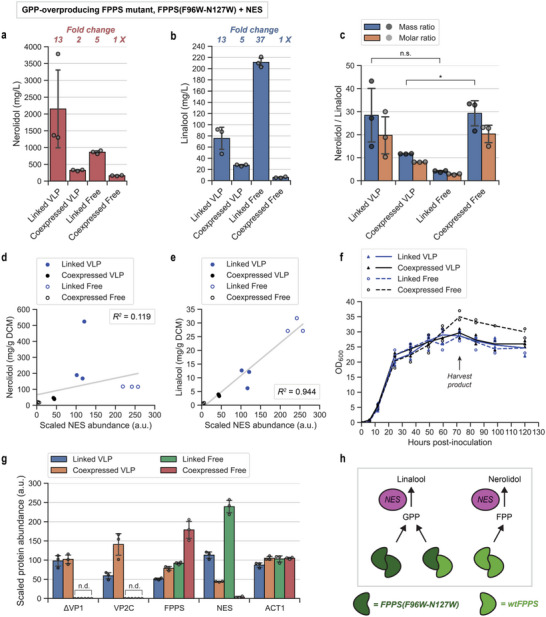
Spatial organization of a GPP‐overproducing FPPS variant (F96W‐N127W) and NES. a) Nerolidol titers at 72 h. “Fold change” indicates the relative titer compared to the Coexpressed Free strain. b) Linalool titers at 72 h. “Fold change” indicates the relative titer compared to the Coexpressed Free strain. c) Mass ratios and molar ratios of the NES products nerolidol and linalool. Statistical analysis was performed using a two‐sample t‐test (two‐tailed; assuming unequal variances): “n.s.” represents no significant difference; “*” represents *p*‐value < 0.05. d) Relationship between nerolidol production and relative NES expression. Fitting a linear regression model using the least squares method produces *R* ^2^ = 0.119. e) Relationship between linalool production and relative NES expression. Fitting a linear regression model using the least squares method produces *R* ^2^ = 0.944. f) Cell density (OD_600_) over the course of fermentation. g) Relative in vivo levels of key proteins at 72 h. “FPPS” abundance includes both the overexpressed F96W‐N127W mutant and native FPPS, which are indistinguishable due to their high sequence identity. ACT1 (actin) is included as a housekeeping reference protein. h) Heterodimers of FPPS(F96W‐N127W) and wtFPPS produce predominantly GPP, and so do homodimers of FPPS(F96W‐N127W). This means that FPP, and in turn, nerolidol production is mainly supported by homodimers of native FPPS. For each chart, means and individual data points are shown. Error bars are +/− 1 STD. “n.d.” = not detected.

We generated a set of strains where FPPS was replaced with a GPP‐overproducing FPPS variant (F96W‐N127W double mutant^[^ ^53^ ^]^) in the galactose‐inducible cassettes. This variant of FPPS has a much lower affinity for GPP compared to the wild‐type enzyme (K_M_^GPP^ = 27.6 µm vs 0.43 µm, respectively),^[^ ^53^ ^]^ which would enhance NES competitiveness for the GPP pool and is expected to increase linalool titers. This was indeed the case (**Figure** **3**b), but with nerolidol titers still being an order of magnitude higher (Figure 3a). The propensity for nerolidol production could be explained by the strong preference of NES for FPP compared to GPP, with k_cat_/K_M_ values of 300 s^−1^ mm ^−1^ for FPP versus 69 s^−1^ mm ^−1^ for GPP.^[^ ^41^ ^]^ It is important to note that the amount of FPP produced by the F96W‐N127W mutant is unknown and, in any case, nerolidol production would also be supported by FPP production by native FPPS. Another factor that could explain the disparity between detected nerolidol and linalool titers is the higher volatility of linalool, which could have led to the greater loss by vaporization.

Each spatial organization strategy led to clearly distinct product profiles, with Linked Free exhibiting a particularly low nerolidol:linalool ratio (Figure 3c). Intriguingly, VLP compartmentalization produced opposing effects on the product profile of NES depending on the co‐encapsulation strategy. The Linked VLP configuration increased the nerolidol:linalool ratio >6‐fold relative to Linked Free (Figure 3c), similar to what was observed with wtFPPS (Figure 2e). On the other hand, the Coexpressed VLP configuration reduced the nerolidol:linalool ratio to ≈0.4 that of Coexpressed Free (Figure 3c). The unusual variability in the product titers of Linked VLP means that the difference between the Linked VLP and Linked Free means was non‐significant; we are unable to explain this variability as proteomics and quantitative PCR did not show signs of multicopy gene integration (Figure 3g; Figures S11 and S12, Supporting Information). Like in the wtFPPS‐overexpressing strains, the Linked Free configuration produced the highest linalool, at 211 mg L^−1^. This is comparable to the current highest reported linalool titer using shake flask culture, which is 219.1 mg L^−1^.^[^ ^54^ ^]^

Metabolite production and intracellular protein levels of the FPPS(F96W‐N127W) strains resembled the trends observed for the wtFPPS‐overexpressing strains. While there was an increase in nerolidol production with higher NES levels, it was not a clear correlation (Figure 3d). In contrast, linalool production increased proportionally with NES expression levels (Figure 3e). As before, NES accumulation was greatly enhanced by enzyme fusion and/or VLP encapsulation, and NES accumulation was highest in Linked Free, followed by Linked VLP, Coexpressed VLP, and Coexpressed Free (Figure 3g). In addition, the FPPS expression of Coexpressed Free was close to double that of the other strains (Figure 3g). Paired with the low NES expression of Coexpressed Free, the high FPPS:NES ratio could have plausibly increased the nerolidol:linalool ratio.

Furthermore, we cannot rule out any additional effects of each spatial organization approach on the local physicochemical environment of the enzymes, such as the enzyme steric environment and metabolite exchange across the compartment shell. In another protein compartment, enzyme encapsulation can result in a change in K_M_.^[^ ^55^, ^56^ ^]^ A high degree of molecular crowding was found to decrease the turnover number (k_cat_) of alcohol dehydrogenase in a bacteriophage P22 nanoreactor, possibly by restricting enzyme dynamics.^[^ ^57^ ^]^ The lipophilicity and charge of the products/substrates could have also influenced their diffusion or introduced nonspecific interactions with the compartment structure – further *in silico* and in vitro studies would be useful to characterize metabolite diffusion across the MPyV VLP in detail.

No obvious growth impairments were exhibited by any of the strains expressing FPPS(F96W‐N127W) and NES (Figure 3f). In the event of a metabolic flux bottleneck, strains overexpressing wtFPPS are expected to accumulate higher intracellular concentrations of FPP than strains overexpressing the GPP‐overproducing variant. We thus inferred that – at least in this strain background – FPP accumulation poses a higher metabolic burden than GPP accumulation.

A potential side effect of enzyme spatial organization is the sequestration of mutant FPPS from native FPPS. Yeast FPPS is predicted to be homodimeric, based on homology modeling and comparisons with the structure of avian FPPS.^[^ ^53^ ^]^ FPPS(F96W‐N127W) subunits are dominant negative, where heterodimerization with a wtFPPS subunit causes the complex to behave like the mutant version and overproduce GPP^[^ ^53^ ^]^ (Figure 3h). Therefore, GPP production is supported by FPPS(F96W‐N127W) homodimers as well as the heterodimer with native FPPS; this is consistent with the high linalool production in the case of Linked Free. Crucially, VLP encapsulation may inhibit the formation of FPPS(F96W‐N127W) heterodimers with native FPPS, thus facilitating FPP production by native FPPS and, in turn, nerolidol production. The ability to promote or impede specific protein–protein interactions using artificial scaffolds could have interesting implications for regulating enzyme behavior in engineered metabolic networks.

## 3 Conclusion

In the field of metabolic engineering, spatial organization is often applied to favor a particular pathway when a single substrate is utilized by multiple enzymes. The novel insight generated from this work is that spatial organization can also be useful when a single enzyme can convert multiple substrates. Moreover, we have provided synthetic biology tools for examining enzyme behavior under different spatial arrangements and enabling a bias toward a desired product. We have thus demonstrated the viability of enzyme spatial organization as a route for tuning the substrate preference of promiscuous enzymes, presumably via altering the accessibility of an intermediate metabolite.

We first extended the MPyV VLP platform for dual protein co‐encapsulation, verifying protein co‐localization using fluorescent proteins. VLP co‐encapsulation and/or translational fusion were used to spatially organize two sequential enzymes in a heterologous biosynthesis pathway, including a promiscuous nerolidol synthase that produces the terpenoid end products nerolidol and linalool. The distinct product profiles resulting from each enzyme configuration suggest that spatial organization can shift the substrate preference of a promiscuous enzyme, although the exact mechanism is not yet understood. Notably, the enzyme does not need to be physically sequestered from non‐target metabolites to create the desired effect. This approach has industrial potential for obtaining high‐purity product directly from the fermentation, as illustrated by Coexpressed VLP (wtFPPS) that exhibited much higher nerolidol production relative to unorganized enzymes but produced virtually no linalool.

We hope to inspire more research investigating the spatial organization of other promiscuous enzymes, particularly terpene synthases. Although metabolon formation has been proposed as a natural mechanism for regulating promiscuous enzymes, this area is currently understudied due to the transient nature of these complexes and technical challenges in engineering artificial metabolons. Overall, our work highlights the utility of synthetic scaffolds as tools for interrogating fundamental questions in biocatalysis, and further establishes the modular MPyV compartment as a versatile platform for in vivo enzyme compartmentalization.

## 4 Experimental Section

### Molecular Cloning and Strain Generation

All cloning was performed by isothermal assembly (NEBuilder HiFi DNA Assembly Master Mix, NEB #E2621), using the plasmid ΔVP1 + VP2C‐GFP^[^ ^11^ ^]^ as the backbone vector. The GFP variant used for this study was yeast‐enhanced GFP (yeGFP3^[^ ^58^ ^]^/GFPmut3^[^ ^59^ ^]^). The mRuby3‐only VLP control (P_GAL1_‐ΔVP1 + P_GAL10_‐VP2C‐mRuby3) was generated by directly replacing the GFP sequence with that of mRuby3.^[^ ^60^ ^]^ The GFP gene was excised from the vector by double digesting with BamHI and BglII followed by gel purification of the backbone. The sequence for mRuby3 was PCR‐amplified from a synthetic gene (synthesized by Integrated DNA Technologies Inc.) to add the appropriate 5′ and 3′ overlapping sequences and assembled together with the linearized backbone vector. For fusion constructs (containing GFP‐mRuby3 or FPPS‐NES), dsDNA fragments were generated of each fusion partner with a short connecting linker peptide (RSAGGGGTGGAEL) added using PCR primer overhangs. The two fragments were then co‐assembled with the linearized backbone vector. For the Coexpressed VLP construct (P_GAL1_‐ΔVP1 + P_GAL10_‐VP2C‐GFP + P_GAL7_‐VP2C‐mRuby3), VP2C‐GFP and T_GAL10_‐P_GAL7_ fragments were amplified from the GFP VLP plasmid and yeast genomic DNA respectively. The two fragments were then co‐assembled with the mRuby3‐only VLP plasmid linearized by digestion with ClaI. The Linked VLP and Coexpressed VLP constructs for nerolidol production were cloned using similar strategies. The FPPS(F96W‐N127W) constructs were generated by excising wtFPPS from each corresponding NES cassette by double digesting with BamHI and BglII, and then cloning in a FPPS(F96W‐N127W) fragment with the appropriate overhangs. Yeast strain genotype details, PCR primers, and synthetic gene sequences are provided in Tables S2–S4 (Supporting Information) respectively. The isothermal assembly and yeast transformation procedures are as previously reported.^[^ ^11^ ^]^ For nerolidol bioproduction experiments, three clones (individual colonies recovered from the transformation) from each construct were maintained as biological replicates. Strains were cultured overnight in YPD medium and stored as 20% v/v glycerol stocks at ‐80 °C.

### VLP Expression, Purification, and Characterization

GFP and mRuby3 particles were expressed and purified as previously described,^[^ ^11^ ^]^ with an additional step prior to polishing by size‐exclusion chromatography: mRuby3‐containing VLP samples (collected after iodixanol cushion ultracentrifugation) were incubated at 37 °C for ≈15 h to allow complete fluorophore maturation of mRuby3. Color change of the samples from colorless/pale yellow to pink could be seen by the eye. Transmission electron microscopy and nanoparticle tracking analysis were performed as described in the previous work.^[^ ^11^ ^]^

### Förster Resonance Energy Transfer (FRET) Assay

Each sample consists of three technical replicates (200 µL each) in black 96‐well microtiter plates. Purified VLPs were diluted in Buffer A (20 mm MOPS, 150 mm NaCl, 1 mm CaCl, pH 7.8) to ≈10 µg mL^−1^ and measured with a microplate reader (Tecan Infinite 200 Pro M Plex). Settings: excitation wavelength = 450 nm (10 nm bandwidth), emission wavelength = 485–650 nm (step size = 5 nm), gain = 150, number of flashes = 10, integration time = 50 µs. The emission spectra were baseline‐subtracted and normalized to each of their respective maximum peaks.

### Super‐resolution Structured Illumination Microscopy (SR‐SIM)

Glass‐bottom dishes (MatTek #P35G‐1.5‐14‐C) were covered with ≈250 µL 0.1% w/v poly‐L‐lysine solution (ProSciTech Pty Ltd #C500) and left to air dry overnight at room temperature. The coated surface was washed thoroughly with distilled water to remove excess poly‐L‐lysine and air‐dried again. Purified VLP samples were diluted to ≈1 µg mL^−1^ in glycerol buffer (90% v/v glycerol, 20 mm Tris‐Cl, pH 8.4). 200–300 µL of diluted sample was carefully settled on the coverslip, tilting the dish slightly to allow the viscous sample to flow and create an even layer. Dishes were prepared at least a few hours to a few days before imaging (stored in the dark at 4 °C) to allow time for the particles to adhere to the coverslip.

Imaging was performed with a Zeiss ELYRA PS.1 equipped with an alpha plan‐apochromat 100 × /NA 1.46 oil immersion objective and an imaging chamber set at 30 °C. Acquisition settings: 3D SR‐SIM mode (11 slices, 0.101 µm interval); five rotations per slice; 1280 × 1280 px; 488 nm excitation/495‐550 nm emission, 30% laser, 100 ms exposure (GFP); 561 nm excitation/570‐620 nm emission, 30% laser, 100 ms exposure (mRuby3). After placing each new dish, temperature was allowed to re‐equilibrate for several minutes before imaging. Sample focusing was performed using the GFP channel, working as fast as possible and moving to a new spot every time to minimize photobleaching. A large z‐interval was required to cover the full range of both color channels in order to account for chromatic aberration and slight variations in the glass surface. SR‐SIM image reconstruction settings: Noise filter = −6, Baseline = shifted, Use raw scale (ScaleToFit = False), PSF = Theoretical, Sectioning = 100, 83, 83. Channel alignment was then applied individually to every processed image (only in Linked and Coexpressed datasets – the mixed samples did not have enough signal overlap to enable automatic alignment). Alignment settings: Fit, Affine. The dataset contains four images for Linked, six images for Coexpressed, and five images for the Mixed control. The number of included particle ROIs is indicated in each plot. Reconstructed and aligned image files were batch‐analyzed with ImageJ (v1.52n) macro scripts. The detailed analysis workflow and ImageJ macro scripts are provided in Supporting Information.

### Nerolidol/Linalool Production

A two‐phase galactose fermentation protocol was used, using dodecane as an inert organic overlay for trapping nerolidol, linalool, and other volatile terpenoid pathway metabolites. Strains were pre‐cultured overnight in YPD medium and inoculated at OD_600_ = 0.05 into 20 mL of a galactose‐containing rich medium (2% w/v Bacto peptone, 1% w/v Bacto yeast extract, 2% w/v galactose, and 0.5% w/v glucose) to initiate the fermentation. The cultures were overlayed with 2 mL sterile dodecane. The methods for flask fermentation, metabolite analysis by high‐performance liquid chromatography (HPLC), and whole‐cell proteomics were as previously described.^[^ ^44^ ^]^

### Data Analysis and Plotting

Charts were plotted using the Matplotlib package in Python 3. Analysis of nanoparticle tracking data was performed using parameters described previously.^[^ ^11^ ^]^ For FRET emission spectra and SR‐SIM histograms, data was smoothed using a Savitzky‐Golay filter with the following parameters: window size = 9, polynomial order = 3 for FRET; window size = 7, polynomial order = 3, binwidth = 0.05 for SIM.

### Statistical Analysis

Linear regressions were fitted using the least squares method with the linregress function in the Python 3 scipy.stats package. Microsoft Excel was used to perform two‐sample t‐tests (two‐tailed; assuming unequal variances) to determine if there were significant differences between groups.

## Conflict of Interest

The authors declare no conflict of interest.

## Author Contributions

L.C.C., F.S., and C.E.V designed experiments. L.C.C. conducted experiments. L.L. performed whole‐cell proteomic analysis by LC‐MS/MS. M.R.P. performed metabolite analysis by HPLC. B.P. developed the base strain for nerolidol production and assisted with experimental design and data interpretation. Z.L. assisted with proteomics and metabolomics experiments. F.S., C.E.V., and G.S. supervised the project. L.C.C., F.S., C.E.V., and G.S. wrote the initial manuscript draft. All authors read and approved the final manuscript.

## Supporting information

Supporting Information

## Data Availability

Additional data, strain information, and a detailed explanation of the SR‐SIM analysis workflow are provided in the Supporting Information file (PDF). Plasmids used in this work are available from Addgene – refer to the Supporting Information file for accession codes. Additional files (including ImageJ scripts and raw microscopy images) can be accessed from UQ eSpace at: https://espace.library.uq.edu.au/view/UQ:eab4972, https://espace.library.uq.edu.au/view/UQ:cf92aa5.
